# Pushing the Limits of Minimally Invasive Spine Surgery—From Preoperative to Intraoperative to Postoperative Management

**DOI:** 10.3390/jcm13082410

**Published:** 2024-04-20

**Authors:** Peter N. Drossopoulos, Arnav Sharma, Favour C. Ononogbu-Uche, Troy Q. Tabarestani, Alyssa M. Bartlett, Timothy Y. Wang, David Huie, Oren Gottfried, Jeanna Blitz, Melissa Erickson, Shivanand P. Lad, W. Michael Bullock, Christopher I. Shaffrey, Muhammad M. Abd-El-Barr

**Affiliations:** 1 Division of Spine, Department of Neurosurgery, Duke University, Durham, NC 27710, USA; arnav.sharma@duke.edu (A.S.); troy.tabarestani@duke.edu (T.Q.T.); christopher.shaffrey@duke.edu (C.I.S.);; 2 Department of Anesthesiology, Duke University, Durham, NC 27710, USAwilliam.bullock@duke.edu (W.M.B.); 3 Division of Spine, Department of Orthopedic Surgery, Duke University Medical Center, Durham, NC 27710, USA

**Keywords:** minimally invasive, spine, TLIF, robotics, ERAS, awake spine surgery, instrument navigation

## Abstract

The introduction of minimally invasive surgery ushered in a new era of spine surgery by minimizing the undue iatrogenic injury, recovery time, and blood loss, among other complications, of traditional open procedures. Over time, technological advancements have further refined the care of the operative minimally invasive spine patient. Moreover, pre-, and postoperative care have also undergone significant change by way of artificial intelligence risk stratification, advanced imaging for surgical planning and patient selection, postoperative recovery pathways, and digital health solutions. Despite these advancements, challenges persist necessitating ongoing research and collaboration to further optimize patient care in minimally invasive spine surgery.

## 1. Introduction

The past several decades have witnessed a tremendous shift towards minimally invasive exposures and techniques, which have enabled surgeons to perform operations while preserving a patient’s natural anatomy and minimizing the total procedural footprint. Spine surgery has been no exception, where advances in surgical technique, intraoperative navigation, surgical instruments, and anatomic visualization have allowed surgeons to perform complex spinal procedures through progressively smaller incisions, using soft tissue sparing techniques. Minimally invasive approaches have quickly gained market share and garnered the attention of both surgeons and patients alike due to their association with reduced blood loss, tissue dissection, operative time, opioid consumption, and earlier hospital discharge [1,2]. While one of the main, ongoing limitations of minimally invasive approaches has been the lack of adequate intraoperative visualization, the introduction of image segmentation and robotics has helped overcome this obstacle by facilitating the accurate placement of instrumentation and avoidance of critical structures [3,4].

Improvements in perioperative care have mirrored advances within the operating room, and helped optimize patient selection, management of comorbid conditions, and return to baseline functional status following surgery. Measures ranging from enhanced recovery after surgery (ERAS) protocols to digital health solutions, like smart phone applications and wearable medical devices, reflect an increasing emphasis on the postoperative management of patients undergoing minimally invasive spine surgery [5,6]. 

This best practice overview aims to describe several categories of notable advancements in minimally invasive spine surgery and highlights several gaps in the literature. Moreover, we aim to encompass the entire patient experience: preoperative optimization, intraoperative adjuncts, and postoperative care pathways, which have all been implemented in the quest to ‘minimize the surgical footprint’ and ensure the best care for spine patients.

## 2. Preoperative Optimization and Artificial Intelligence-Informed Risk Calculations

Given the comorbid conditions affecting the aging United States population, care should be taken to preoperatively optimize these patients in an effort to decrease postoperative morbidity. Although several studies describe better outcomes following stricter preoperative management of specific diseases, there is no universally accepted pathway or protocol to preoperatively evaluate for comorbid conditions, which are likely to complicate the perioperative course [7,8].

For example, the increased prevalence of osteoporosis-spectrum disorders rises with age and poses unique challenges to the spine surgeon [9]. Compromised bone mineral density may be evaluated through DEXA scans, CT-based Hounsfield unit measurements, and MRI scoring systems [9]. If bone mineral density compromise is identified, patients may be preoperatively treated with medications to augment bone density [10]. Further, early identification of bone mineral density is critical as there are specific instrumentation changes a spine surgeon may make to combat decreased screw purchase, like cement augmentation or expandable pedicle screws [11,12]. Osteoporosis is just one example of the many comorbid conditions common in spine patients. Recently, several studies have attempted to define preoperative care pathways to holistically evaluate the preoperative spine patient, identify comorbid conditions that are actionable, and inform surgical planning [13,14,15,16].

### 2.1. Big Data

The integration of large data sets and institution-specific analyses to offer patient-specific insights like risk stratification and patient selection is adding new value to preoperative planning. Algorithms such as traditional regression modeling and advanced algorithms like decision trees, support vector machines, and neural networks, have shown promise in accurately predicting surgical outcomes [17]. By quantifying patient risk factors, like comorbid conditions and overall health status, these tools enable a detailed discussion of potential surgical risks and the anticipated timeline for postoperative recovery. However, we must avoid complete reliance on such tools. Their purpose is to inform, not dictate, surgical decision making; further, these technologies are intended to complement, not replace the spine surgeon’s expertise and objective clinical findings. Cautious, balanced integration holds the potential to refine surgical planning and enhance the overall quality of spine care.

Several of the largest database studies to date use the data contributed to the National Surgical Quality Improvement Program (NSQIP) [18]. Unfortunately, this dataset does not offer detailed information particularly germane to spine surgery. Consequently, parameters that may significantly improve the models are not captured by the dataset. There are, however, many commendable, large-scale studies that offer important insights that draw from this dataset. For example, in their cohort of nearly 300,000 spine surgery patients, Cole et al. found that interhospital transfer was a strong independent predictor for worse postoperative outcomes among spine surgery patients [19]. They additionally found that the frailty score, a quantification of physiological reserve, of transferred patients harnessed the greatest discriminative ability for poor postoperative outcomes [19]. One interesting qualitative study on NSQIP risk score usage found that over half of the surveyed surgeons used this risk score calculator in fewer than 20% of their preoperative discussions with patients. However, use was higher among older, functionally dependent patients undergoing nonelective cases [20]. Respondents of this survey cited concerns over the calculator’s accuracy and barriers to use as the leading reasons for the lack of adoption into regular preoperative workflow [20].

Specific to spine surgery, there is one predictive model, SpineSage, created from a registry of approximately 1500 spine surgeries which set out to predict major postoperative medical complications [14]. The final published model had a receiver operator curve (ROC) characteristic of 0.76 for predicting any complication and an ROC characteristic of 0.81 for predicting major complications [14]. In a follow-up study of 315 patients undergoing spine surgery, Coia et al. found that NSQIP, compared to SpineSage, severely underestimated ‘any complication’ (SpineSage: predicted 30%, NSQUIP: predicted 8%, observed risk: 35%) [21]. Several other studies have similarly found that the NSQIP risk calculator underperforms on specific cohorts of preoperative spine patients and is not very generalizable to this patient population [15,16].

#### Machine Learning

Machine learning (ML) algorithms are increasingly being recognized for their potential to revolutionize spine surgery, offering new avenues for improving patient outcomes and selection. By analyzing vast datasets, ML can uncover patterns that may assist in preoperative planning, risk stratification, and postoperative care. This innovative approach may enhance the surgical decision-making process and personalize patient care, setting a new standard in spine surgery.

Several recent studies have deployed machine learning algorithms for the prediction of prolonged opioid prescription, mortality, and discharge modality. In one large study of almost 60,000 spinal fusion cases from the NSQUIP database, Goyal et al. deployed several ML algorithms in an effort to predict nonhome discharge [22]. All classification algorithms showed excellent discrimination for this prediction, achieving areas under receiver operating characteristic curves of greater than 0.8. An interesting study, however, for such algorithms to be clinically translatable, they must offer actionable insights. Accordingly, Karhade et al. have developed several web-applications for predicting postoperative mortality following operative management for metastatic spinal disease and prolonged postoperative opioid prescription [23,24]. In these cases, patients who are found to be at high risk of mortality following metastatic spinal disease treatment may be reconsidered for the appropriateness of a potentially futile treatment. Moreover, should patients be identified as at higher risk of extended opioid use, various changes may be made to the perioperative analgesic regiment, which we will later discuss.

Recently, Corey et al. described a novel machine-learning tool named Pythia, being used to identify high-risk surgical patients from 194 clinical features [13]. This tool used local electronic health record data, comprising 90,145 patients who underwent 145,604 invasive procedures, which led to the creation of a 9-question calculator to produce a risk assessment for 14 general categories of postoperative outcomes. Ultimately, the Pythia model’s sensitivity and specificity were 76% in identifying high-risk (greater than 15% chance of any complication) patients [13]. Briefly, at our institution, we use this tool to preoperatively risk stratify patients into low-, medium-, and high-risk cohorts for postoperative complications. Should a patient score into the medium- or high-risk cohort, they are subsequently referred to, and evaluated by, a dedicated preoperative optimization clinic based on their specific needs. The novelty of this model precludes large-scale prospective evaluation—more work is required before we may characterize the value of such a workflow and recommend other institutions adopt similar means should they have the resources to do so.

## 3. Advanced Imaging-Based Surgical Planning

Wilhelm Conrad Roentgen’s groundbreaking discovery of X-rays in 1895 marked the start of the advanced imaging era. Unbeknownst to this pioneer, his work would profoundly transform the landscape of surgical planning. Since this initial inroad, the field has undergone exponential growth; stepwise iteration has led to the creation of advanced imaging modalities including computed tomography (CT) and magnetic resonance imaging (MRI). These advancements have enabled accurate visualization of the intricate osseous, soft tissue, and neurovascular structures of the spine. From the initial description of the X-ray to the multiplanar images that are now a cornerstone of spinal diagnostics and surgical planning, continued evolution will cement these modalities as indispensable tools for all spine surgeons alike. In this section, we will explore one of the most recent adaptations of advanced imaging-informed surgical planning in spine surgery, three-dimensional neurosegmentation.

Accompanying the progression of surgical techniques for access to the spine in an effort to optimize surgical planning, advanced imaging-assisted preoperative planning via three-dimensional spine segmentation has massed increased attention in the literature. Briefly, using commercially available software (Origin Server 3.4, BrainLab, Munich, Germany), relevant pathologic levels are manually traced on T2-SPACE-weighted MRI imaging using the software’s native region-growing selection algorithm [25]. Initially, this technology allowed for enhanced visualization of pathologic anatomy; it has since been adapted to inform surgical decision making [25]. Tabarestani et al. showcased the first adaptation of this technology in preoperative planning for minimally invasive lumbar interbody fusion [25]. In this study, the team segmented the transfacet and trans-Kambin’s triangle corridors to define key anatomical landmarks and inform maximal cannula diameter sizes. Accordingly, future patients who are candidates for transforaminal interbody fusion may have their anatomy segmented to inform optimal access technique and laterality. Ultimately, this demonstrates a shift to a more nuanced, patient-specific approach to minimally invasive spine surgery. 

It is important to note that this technology carries notable limitations. At this time, three-dimensional segmentation is a tedious, manual process that may take up to an hour per patient. For this reason, in urgent or trauma cases, delaying care to use this technology is not reasonable. Accordingly, there is a significant effort to develop artificial intelligence-based automatic segmenting protocols, though they are largely targeting vertebral segmentation [26]. Once validated, this technology has the potential to substantially impact patient care and favorably disrupt patient selection and surgical planning for minimally invasive spine surgery patients. Below, the authors present our institutional experience of using preoperative 3D neurosegmentation to identify the optimal approach technique and laterality for a patient.

### Representative Case

**Figure 1:**
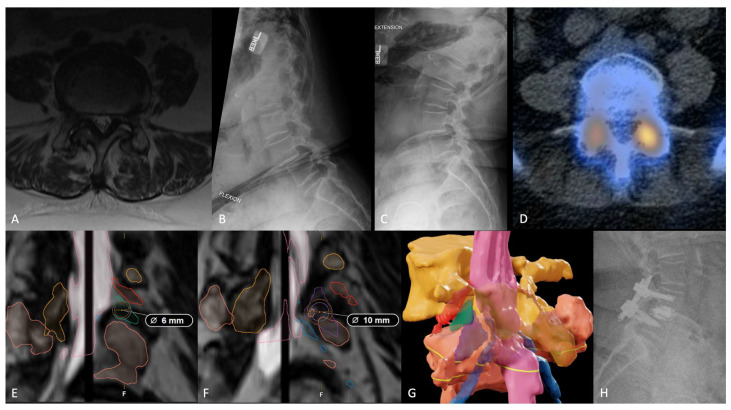
Axial T2-weighted MRI slice at L4–L5 (**A**) demonstrating severe bilateral lateral recess stenosis. Flexion (**B**) and extension (**C**) plain films demonstrating a grade 1 spondylolisthesis at L4–L5. CT-SPECT (**D**) depicting increased radiotracer uptake at the bilateral L4–L5 facet joints, left greater than right. Neurosegmentation of the left trans-Kambin’s triangle corridor ((**E**), green triangle), offering a maximum cannula diameter of 6 mm. Segmentation of the left transfacet corridor ((**F**), purple triangle), offering a maximum cannula diameter of 10 mm. Three-dimensional neurosegmentation of L4–L5 (**G**) showing the thecal sac (pink), left Kambin’s triangle (green triangle), left transfacet corridor (purple triangle), L4 nerve root (red), and bilateral L5 nerve roots (blue). Postoperative upright lateral lumbar plain film (**H**) demonstrating adequate hardware placement.

Case 1 is a 67-year-old female who presented with lower back and left hip pain that radiated down her bilateral lower extremities to the level of her ankles for several months without any precipitating events. Subsequent evaluation with MRI (Figure 1A) and flexion-extension plain films (Figure 1B,C) demonstrated a grade 1 L4/5 spondylolisthesis causing severe bilateral foraminal stenosis. Subsequent CT-SPECT corroborated her pain distribution and presentation, demonstrating increased radiotracer uptake foci at the L4–L5 level, left greater than right (Figure 1D). Conservative therapy with gabapentin, steroid taper, physical therapy, and CT-guided transforaminal epidural steroid injection at this level provided significant but temporary relief for 2 months. Her symptoms greatly affected her quality of life and her ability to complete activities of daily living.

Given her presentation, we planned for instrumented fusion via transforaminal lumbar interbody fusion (TLIF). In an effort to offer this patient the best, anatomy-specific, TLIF technique, we segmented this patient’s spine (BrainLab, Munich, Germany) to delineate key surgical anatomy. Segmenting out the anatomic borders, and defining the different approach corridors bilaterally, allowed us to determine the optimal approach and laterality specific to this patient’s anatomy. Three-dimensional neurosegmentation revealed left-sided volumes of transfacet (TF) corridor, 131.9 mm^2^ (allows for a 10 mm cannula, Figure 1F) and trans-Kambin’s (TK) triangle corridor, 56.7 mm^2^ (allows for a 6 mm cannula, Figure 1E). Right-sided volumes were both smaller, TF: 125.5 mm^2^ and TK: 112.5 mm^2^, Figure 1E. Given her left-greater-than-right CT-SPECT radiotracer uptake, and the larger cannula diameter facilitated by the left transfacet corridor, we ultimately offered and proceeded with a left-sided minimally invasive TF-TLIF, accessing the more anatomically favorable side. Following interbody insertion, both facets were decorticated, and an allograft was placed. Finally, rods were inserted along the previously inserted pedicle screws bilaterally. 

The case was completed without complication, quiet electromyography, 50 mL estimated blood loss, and the postoperative course was largely uneventful. The patient was discharged home on postoperative day 2. By the 3-month follow-up visit, her preoperative pain and radiculopathy had almost completely resolved. Oswestry Disability Index improved to 26 from 62. Visual analog pain scale (VAS, back) improved from 5 to 0, and VAS (leg) improved from 9 to 1.

## 4. Regional Anesthesia and Awake Spine Surgery

In contrast to the systemic effects of general anesthesia (GA), regional anesthesia (RA) offers targeted pain relief to the body regions defined by the injection site. Extensively discussed in the orthopedic literature, regional anesthesia may work synergistically with GA to provide intraoperative and postoperative analgesia to patients and mitigate opioid use [27]. Specifically in spine surgery, neuraxial and paravertebral RA techniques provide focused analgesia and have garnered increased attention recently for their benefits in mitigating the well-described morbidities of GA use alone [28,29,30]. In this section, we aim to explore the application of supplemental RA use in spine surgery and describe the accompanying benefits and indications that ultimately made awake spine surgery possible.

Neuraxial RA use in spine surgery comprises spinal (SA) and epidural anesthesia (EA), though spinal anesthesia is the more common modality [31]. SA is administered directly into the cerebrospinal fluid. It is typically administered in a sitting or lateral decubitus position at the respective spinal level, generally below L2 to avoid spinal cord damage, after which the patient is turned supine for a few minutes, allowing the block to settle. Desired sensory levels typically range from T6 to T10 and the onset of medications through SA takes approximately 5 min or less. After this, the patient may be repositioned into a sitting, knee–chest, lateral, or prone position per the surgeon’s discretion and the chosen surgery [32]. Anesthetic agents commonly used are comprised of a long-acting local anesthetic such as bupivacaine in combination with fentanyl [32]. 

**Figure 2:**
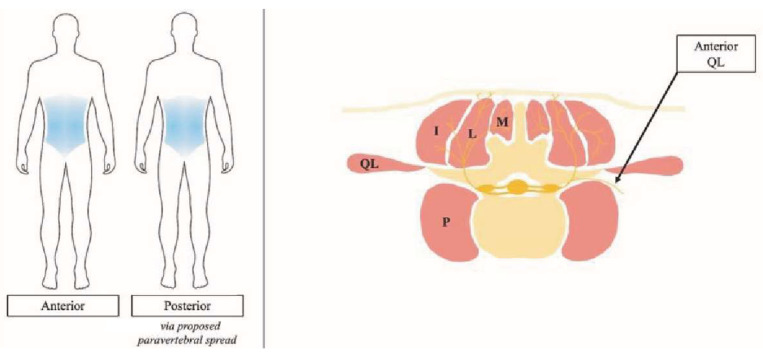
Analgesia distribution, left, and injection site, right, schematic for the quadratus lumborum block. (M: multifidus, L: longissimus, I: iliocostalis). Figure reproduced with permission from [31].

Paravertebral analgesia provided by the paraspinal muscular block during lumbar surgeries, including discectomies, laminectomies, and fusions, is the most frequently described application of RA use, though feasibility has been demonstrated in thoracic cases as well [33]. In such cases, these paravertebral blocks have led to decreased intraoperative and postoperative opioid consumption without unduly burdening the patient with more pain and complications [34]. Interestingly, there is an emerging paravertebral RA technique that uniquely provides circumferential analgesia, Figure 2. The quadratus lumborum (QL) block involves the injection of local anesthetic along the QL via anterior, lateral, and posterior approaches. Together, this provides analgesia along the entire operative scope of an anterior lumber interbody fusion. Although the reports of QL block on circumferential spine surgery patients are sparse, the early available evidence suggests that it is a reasonable way to provide analgesia and mitigate opioid use [35,36]. 

### 4.1. Awake Spine Surgery

Though awake surgeries in neurosurgery have typically been confined to craniotomies, the emergence of RA has expanded the awake surgery scope to include procedures of the spine. Awake spine surgery presents a unique set of benefits compared to traditional general anesthetic approaches, potentially allowing for reduced postoperative hospital stays, in-hospital complications, and cost of surgery while expediting recovery and rehabilitation [32,37]. Since patients are able to consciously protect themselves in positioning, risks associated with prone positions including corneal abrasions, brachial plexus injuries, neck pain, and shoulder pain may effectively be eliminated [32]. 

One of the most important advantages of awake spine surgery is that it may allow patients who would otherwise not be candidates for general anesthesia to be eligible for spine surgery. Specifically, the elderly population, with poor physiologic reserves is often challenged by postoperative cognitive dysfunction [38]. Salven et al. reviewed RA use in spine surgeries with highly comorbid patients, and concluded that the ideal candidate for awake spine surgery under RA may indeed carry one or multiple comorbid conditions [31]. In fact, in a large study of 424 consecutive patients who underwent awake spine surgery, Wang et al. found that for both simple and complex lumbar spine surgeries, SA was safe and feasible in medically complex elderly patients [39]. Specifically, the 46 patients greater than 80-year-old experienced no significant difference in rates of spinal headache, deep vein thrombosis, pneumonia, urinary retention, readmission within 30 days, or acute pain service consult relative to their younger, ‘healthier’ (as measured by American Society of Anesthesiologists score) counterparts [39]. A similar conclusion was reached in a retrospective analysis of 146 ASA II–III patients undergoing lumbar surgery, in which SA safely provided analgesia in high-risk cardiovascular patients with stable hemodynamics at a potentially lower cost [40]. Taken together, SA serves as a tool to provide safe and effective analgesia to patients who may be precluded from surgery due to the well-described constraints and morbidities of GA. Certainly, SA requires further evaluation through a randomized prospective study to more completely characterize its safety profile, indications, and most importantly, contraindications.

### 4.2. Awake Spine Surgery Limitations

Though RA shows promise as a safe primary anesthetic approach, there are certainly notable limitations compared to GA. In patients with a higher risk for airway compromise, such as those with obesity or COPD, intraoperative airway patency is more difficult to maintain without intubation and relatively contraindicates awake spine surgery via SA. Moreover, due to the short lifespan of SA, there also exists a limit on surgical time. SA use alone for awake spine surgery generally offers a limited time frame and is optimal for procedures lasting less than 4 h, after which SA wears off, cephalad to caudad [41]. Therefore, it is inherently more suited for simple and limited procedures [42]. Interestingly, however, one 343-case retrospective chart review explored the viability of SA for complex lumbar surgeries across a broad spectrum of age and health statuses and found no significant difference in complications versus GA [43]. As such, evidence has continued to support RA as a potentially more efficacious modality for short lumbar operations, but there exists potential for RA use in more complex cases as well. 

### 4.3. Representative Case

**Figure 3:**
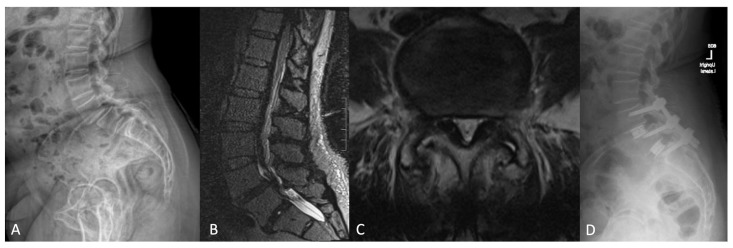
Cropped section of a preoperative lateral upright whole spine film (**A**) demonstrating the spondylolisthesis at L4–L5 and L5–S1. Preoperative sagittal (**B**) and axial (**C**) T2-weighted MRI slices demonstrating severe central canal and lateral recess stenosis, ligamentum flavum hypertrophy, and diffuse disc bulge. Postoperative upright lateral lumbar spine plain film (**D**).

Below, the authors share their awake spine surgery experience through an illustrative case. Case 2 is a 74-year-old female who presented with lower back and bilateral leg pain for 7 years. In the interim, she had exhausted all conservative therapy measures, and her pain severely limited her quality of life. Subsequent evaluation with upright whole-spine films (Figure 3A) and MRI (Figure 3B,C) revealed grade 1 spondylolisthesis of L4–L5 and L5-S1, ligamentum flavum hypertrophy, and significant central and foraminal stenosis at these levels. Given these findings, we ultimately offered the patient an L4–S1 minimally invasive, percutaneous, interbody fusion with posterior instrumentation.

Around the same time the operating room was reserved for this procedure, our institution halted all elective surgeries in light of the COVID-19 pandemic in an effort to ensure the proper allocation of resources on other hospital units. We were only able to proceed with case scheduling if the patient would be safely discharged home from the post-anesthesia care unit. Accordingly, we offered and proceeded with an awake, CT-navigated, L4–S1 percutaneous TLIF under spinal anesthesia. 

The uncomplicated case was completed in under 3 h, with an estimated blood loss of less than 50 mL, and the patient was discharged home the same day after voiding, eating, and ambulating. At this time, during a 2-year postoperative follow-up visit, she reported significant improvement in all symptoms. VAS leg and back both improved from 7 to 1 and her ODI improved from 70 to 10. Through this case, we attempt to illustrate the adaptability of awake spine surgery.

## 5. Intraoperative Advances—Fluoroscopic Navigation, Robotics, and Endoscopy

The advent and widespread adaptation of MIS surgical techniques has revolutionized the field of spine surgery, offering patients and surgeons less-tissue-destructive alternatives. Importantly, the growing body of literature corroborates the maintenance of fusion rates and outcomes while retaining a commendable safety profile [44,45,46]. Coinciding with the rise of MIS techniques and adaptations, there has been marked advancement in intraoperative imaging to maintain an appropriate and safe degree of visualization. Accordingly, the field of spine surgery has observed incremental improvement in methods to more safely and accurately navigate instruments and reduce radiation exposure compared to traditional MIS methods.

### 5.1. Fluoroscopic Navigation

The principal challenge inherent in MIS surgery imaging stems from the reduced direct visualization of the patient’s anatomy. This necessitates the use of imaging systems to facilitate safe instrumentation and introduces a notable limitation: radiation. The radiation burden is two-fold, (1) radiation exposure of the surgeon and operative team, and (2) radiation exposure of the patient—this cumulative radiation exposure is clinically significant. In a large cohort study of female surgeons in various surgical subspecialties, Chou et al. found that the orthopaedic surgeon cohort experienced almost twice the prevalence of any-cancer, and strikingly, almost three times the expected prevalence of breast cancer [47]. Similarly, for patients, a large metanalysis revealed a 2.4-fold increase in radiation dose to the patient during MIS-TLIF compared to open fluoroscopic-assisted TLIF [48]. Taken together, the importance of radiation-reduced methods during MIS-surgery visualization cannot be overstated. Advancements in navigated spine surgery technology have addressed each of the aforementioned radiation burdens (1) and (2) in a stepwise fashion.

CT-navigated pedicle screw placement represents the first radiation-reducing advancement over traditional live fluoroscopy. The advent of CT navigation all but eliminated intraoperative radiation exposure to the operative team, at the expense of exposure to the patient [49]. In a prospective study, Bratschitsch et al. found that the surgeon experienced approximately a 10-fold reduction in radiation dose to the right hand and a 5-fold decrease in thoracic radiation exposure when comparing CT navigation to live fluoroscopy, an expected decrease at the expense of a limitation; the operative team vacates the operating room during registration scans and the patient is radiated [50]. Additionally, CT navigation affords enhanced three-dimensional instrument planning over fluoroscopy, which has led to increased pedicle screw placement accuracy [49]. Unfortunately, the enhanced visualization and decreased operative team radiation exposure come at the expense of increased patient radiative burden [50]. 

**Figure 4:**
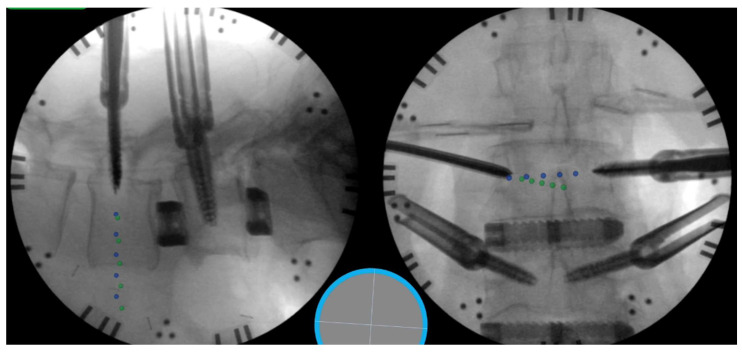
Intraoperative pseudo-live instrument tracking, surgeon’s view (TrackX, Chapel Hill, NC, USA). (**Left** image), lateral projection depicting the trajectory of bilateral pedicle screws (green and blue dotted line). (**Right** image), AP projection of the bilateral pedicle screw trajectories (green and blue dotted line).

Most recently, ‘pseudo-live’ fluoroscopic instrument tracking has emerged, reducing radiation exposure, and simplifying operating room set up over CT navigation (only one standard c-arm is required) [51]. This technology was first described by Wang et al. in a 2019 feasibility study showcasing pseudo-live tracking [51]. Importantly, this technology allows for multi-instrument tracking and screw trajectory visualization, (Figure 4). In a randomized control follow up to their previous study, Wang et al. revealed that this novel instrument navigation technique significantly reduced both radiation exposure (by 83%, *p* < 0.0001) and total procedure time (by 81%, *p* = 0.0003) compared to traditional live fluoroscopy [52]. Similarly, in later, larger studies, Hamouda et al. and Wang et al. corroborated the reduction in radiation exposure, and procedure time [53]. Here, it is important to note that the usage of this technology complements awake spine surgery as the precious analgesic effect of SA is not wasted during the required registration scans of CT-navigated instrumentation. Taken together, these efforts offer a promising avenue for further radiation reduction on patients and members of the operative team while improving operative efficiency.

### 5.2. Robotics

Since the introduction of the Mazor SpineAssist in 2004 as the first spinal navigation robot, the field of robotic spine surgery has rapidly expanded. Today, several multimillion- and billion-dollar companies have invested in the development of robotic spine technology. While studies suggest improved accuracy in pedicle screw placement compared to traditional, free-hand, methods, questions remain regarding the cost-effectiveness, efficiency, and overall clinical outcomes of robot-assisted surgery. As we move through this bourgeoning era of robotic-assisted spine surgery, comprehensive evaluations of these technologies will be essential to fully describe their true value.

Robotic applications in spine surgery are primarily aimed to mitigate pedicle screw malposition and subsequent complications. At the time of writing, there are several randomized control trials that compare pedicle screw placement accuracy to free-hand methods. In a metanalysis of these studies, Peng et al. found that there was no statistically significant difference in pedicle screw placement accuracy between robotic-assisted surgery and traditional methods [54]. Interestingly, however, on subgroup analysis, they found that the TiRobot significantly improved pedicle screw accuracy while the use of the SpineAssist robot was associated with less accurate placement compared to free-hand [54]. It is important to note, however, that the heterogenicity of the included trials was significant [54]. Contrastingly, in subsequent, and more inclusive metanalyses of 1525 patients and 6262 patients, respectively, Fatima et al. (OR: 1.68) and Naik et al. (Odds Ratio: 2.25) found that robot-assisted surgery resulted in more accurate pedicle screw placement than free-hand methods [55,56]. Further supporting the accuracy of robot-assisted surgery, Naik reported a SUCRA hierarchical ranking S-score for robotic-assisted surgery of 93.7% versus that of free-hand surgery at 26.7%, and CT navigation without robotics at 38.6%. Although there appears to be some variability among specific robotic platforms and their individual efficacy, the preponderance of evidence suggests that robotic navigation is associated with increased pedicle screw placement accuracy. 

It is important to recognize several limitations of spine robotics. Like CT-navigated techniques, current robot-assisted surgery platforms require an intraoperative registration CT scan for instrument tracking and trajectory planning, which represents a reversal of efforts to mitigate radiation exposure to the patient. Most importantly, there is some question as to the safety and outcomes of robotic-assisted spine surgery. In a large, matched cohort analysis of 2528 (per cohort) patients, comparing robotic-assisted to conventional lumbar spinal fusion, Yang et al., found that robotic assistance was independently associated with increased risk of revision surgery, infection, instrumentation complications, and postoperative opioid use [57]. Here, the authors propose that increased operative time and robotic experience are large contributing factors to these findings [57]. Moreover, there are conflicting reports as to how robotic spine surgery affects surgical duration with many studies reporting, shorter, longer, or equal times compared to conventional techniques [58,59,60]. More work is certainly required to continuously iterate these robotic technologies with specific effort and emphasis on operative efficiency maintenance and patient safety. Perhaps pseudo-live fluoroscopy may bridge this gap and improve upon potential robotic inefficiency while maintaining an appropriate safety profile.

To this date, there is only one study directly comparing robotic-assisted surgery to pseudo-live fluoroscopy. In this study, Wang et al. report the average operative times for robotic MIS-TLIFs at 175 min, while the same surgery done under pseudo-live fluoroscopy averaged 120 min [53]. Given the high cost of the spine operating room per minute, over the course of a year, repeated cost savings may be translated to the patient and hospital [61]. Furthermore, a surgeon may now have the capacity to perform an additional surgery during the same workday [61]. As the adoption of these novel technologies increases, it is imperative that they are compared to the longer-standing alternatives to ensure that conferred benefits do not come at the cost of patient safety.

### 5.3. Novel Robotic Applications

**Figure 5:**
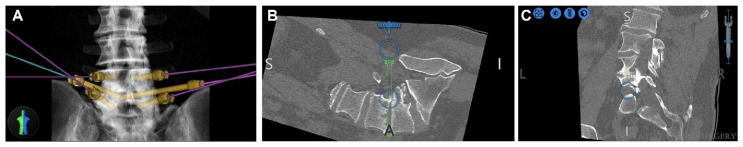
(**A**) Preoperative screw trajectory projections into Kambin’s triangle. (**B**) Sagittal trajectory planned as caudal as possible to avoid L4 nerve root violation. (**C**) Coronal trajectory into disc space at the mid-pedicle level, the largest safe area in Kambin’s triangle. Reproduced with permission from [63].

Finally, there have been several novel reports of robotic utilization across the field. It is perhaps intuitive to understand the benefit conferred by the ultra-precise instrumentation of the spine robot in scenarios requiring disc-space access through the minute corridor of Kambin’s triangle, for example [62]. Here, the traversing nerve root is particularly susceptible to violation considering Kambin’s triangle areas may range as low as 60 mm^2^ [62]. Demonstrating the feasibility of robotic-assisted disc space access through this small corridor, Dalton et al. reported excellent radiographic outcomes and no complications in their case series of 10 patients who underwent robotic-assisted percutaneous lumbar interbody fusion through Kambin’s triangle [63]. Making use of the robot’s enhanced surgical precision, the disc space trajectory may be mapped to Kambin’s triangle while obviating the need for superior articular process drilling and ensuring avoidance of adjacent structures. To our knowledge, this is the first description of such in the literature, Figure 5. Though this is not a previously described application for the spine robot, in patients with marked anatomical constraints or aberrant anatomy, the robot may facilitate safe trajectory planning and select-in more patients for minimally invasive, percutaneous interbody fusion.

**Figure 6:**
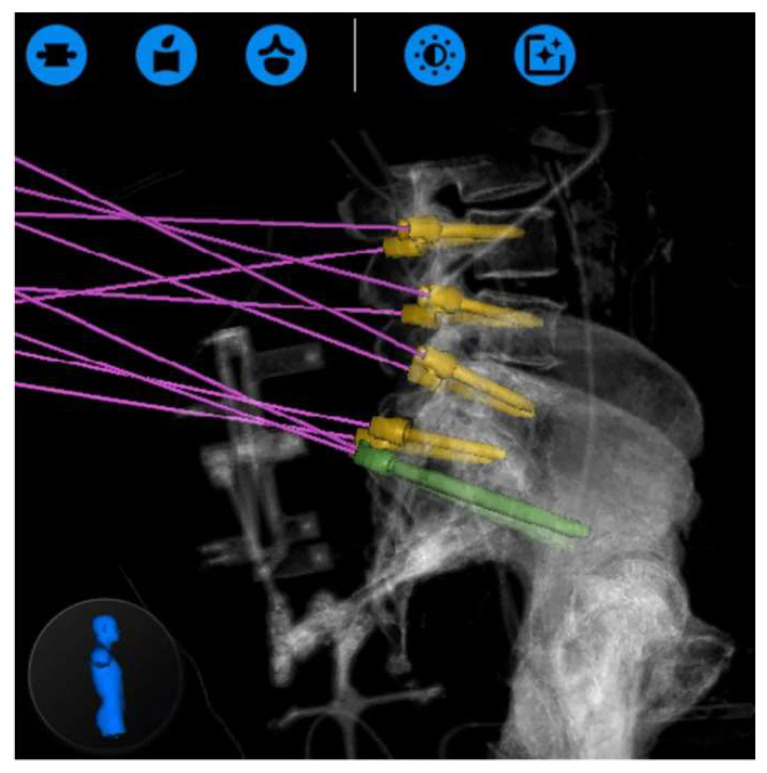
Intraoperative robotic trajectory planning (Globus ExcelsiusGPS^®^, Globus Medical Inc., Audubon, PA, USA) for placement of L3–S1 pedicle screws, yellow, and iliac bolts, green. Reproduced with permission from [67].

**Figure 7:**
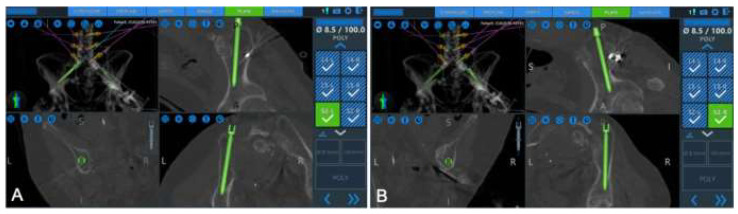
Left (**A**) and right (**B**) iliac bolt placements in line with lumbar pedicle screws. Reproduced with permission from [67].

Another implementation of the spine robot is to facilitate spinopelvic fusion. There are several reports of robotic-assisted S2 alar-iliac (S2AI) screw placement and percutaneous iliac screw fixation. In the largest multicenter study reporting on robotic-assisted S2AI screw fixation, Lee et al. reported no intraoperative complications and a mean overall screw accuracy of 93.8% across 65 screws [64]. Several other groups have reported similar experiences [65,66]. Although the S2A1 screw has several documented advantages over traditional iliac bolts by notably eliminating the requirement of side connectors and additional tissue dissection, cases that involve destructive lesions of the sacrum contraindicate S2A1 instrumentation in favor of traditional iliac screws [67]. Here too, spine robots found application. Park et al. reported a positive, complication-free experience when placing percutaneous iliac screws in patients with gross sacral lesions [67]. By utilizing the advanced trajectory planning of the robot, Park et al. were able to align their iliac instrumentation with the above pedicle screws and avoid placing side connectors, Figure 6 and Figure 7.

### 5.4. Endoscopy

Spine surgery has evolved significantly since the first discectomy was performed in 1908 by Kraus and Oppenheim. These early surgical techniques were plagued by serious complications, which led to relatively poor postoperative outcomes [68]. The development of microsurgical approaches in the late 1970s by Yasargil and Casper marked significant advancement and established a new standard for neurosurgical approaches [69]. Innovation continued which culminated with fully functional endoscopic systems resulting in procedures that offer higher patient satisfaction, reduced lengths of stay, and lower opioid usage while maintaining a high degree of safety [70,71]. At this time, the endoscope may be employed for foraminal and lateral recess stenosis and cases of low-grade spondylolisthesis. However, in cases of high-grade spondylolisthesis or severe bilateral radiculopathy (where contralateral indirect decompression will not correct the pathology), endoscopic surgery is relatively contraindicated. 

As with any new technique or iteration, it is important to characterize endoscopic surgery’s safety and outcome profile. One study compared endoscopic to traditional MIS-TLIF found that the endoscopic adaptation resulted in decreased operative blood loss (*p* < 0.001) [72]. Postoperative low-back and leg pain and Oswestry Disability Index decreased in both groups (*p* < 0.05) [72]. Notably, there were no complications with either group, nor was there any statistically significant difference in fusion rates between cohorts [72]. Other studies, including a large metanalysis, have corroborated these findings [73,74]. Moreover, when compared to traditional MIS-TLIF, endoscopic lumbar interbody fusion has been repeatedly found to result in similar fusion rates [75,76]. More recently, the application of endoscopic techniques to degenerative cervical spine disorders has similarly demonstrated favorable results when compared to traditional techniques [77,78].

Lastly, the same previously described advancements, CT navigation, and robotics, have been applied to endoscopic spine surgery. Endoscopic CT-navigated spine surgery has demonstrated consistently favorable short- and long-term outcomes in the few studies that are available [79,80]. Similarly, robotic-endoscopic applications appear to provide favorable results at present; however, these studies are limited to very small sample sizes and retrospective studies. Specifically, Wang et al. found that robot use in endoscopic lumbar discectomy was associated with less intraoperative blood and similar complication profile [81]. Further study is certainly required to describe this robotic application more completely. 

## 6. Postoperative Advances—Enhanced Recovery after Surgery (ERAS)

### 6.1. General Overview

Finally, we discuss post-surgical advancements. Enhanced recovery after surgery (ERAS) protocols are designed to achieve and optimize early postoperative recovery. The components of ERAS include preoperative counseling, nutrition and fluid optimization, standardized pain management regimens, and early ambulation, which work together to facilitate a safe, expeditious return to baseline [82]. Minimally invasive spine surgery aligns with ERAS protocols by reducing postoperative blood loss, decreasing muscular trauma, accelerating postoperative ambulation, decreasing surgical site infections, and decreasing reliance on opioids [83]. 

ERAS protocols can be broadly divided into preoperative, intraoperative, and postoperative arms. The preoperative arm of ERAS focuses on preparing the patient for the upcoming surgical procedure through the administration of anticipatory analgesia such as gabapentin, pregabalin, or acetaminophen [84]. Additionally, the patient is extensively educated on their anticipated surgery, with appropriate goals and expectation settings. Patient nutrition is evaluated and optimized [84]. The intraoperative arm of ERAS involves management during the index operation and focuses on the judicious use of non-opioid analgesia modalities [84]. Finally, the postoperative arm is dedicated to facilitating early mobility with physical/occupational therapy, early nutrition, wound care, and continued multi-modal pain management with an emphasis on minimizing opioid use [84]. 

### 6.2. Specific Implementation Aims and Outcomes

Current ERAS protocols, though different, maintain commonalities in their means. Postoperative ERAS targets include pain improvement, time to postoperative ambulation, and use of medication including opioids, anti-inflammatory drugs, and muscle relaxants [85]. One of the primary goals behind tailored pain management strategies is to minimize opioid use, especially in the setting of rising opioid overuse [86]. A 2019 study of patients undergoing minimally invasive lumbar decompression surgeries found that patients who were given opioids as part of their anesthesia had significantly elevated rates of opioid consumption postoperatively compared to their ERAS counterparts receiving opioid-free anesthesia [85]. Furthermore, there was no significant difference in post-anesthetic care unit pain scores [85]. Instead of opioids, patients are given other multimodal analgesics including ketamine, intraoperative dexamethasone, ketorolac, local anesthetic, and non-steroidal anti-inflammatory drugs [87]. This largely parallels, and may complement, the previously described advantages of RA. Current research also highlights that ERAS protocol implementation in minimally invasive spine surgery is associated with faster postoperative time to ambulation, which may positively affect postoperative patient morbidity and length of stay. However, this will require a longer-term follow-up study to state with certainty [85,88].

### 6.3. Multidisciplinary Approach

The creation and implementation of ERAS protocols is inherently a multidisciplinary, patient-centered process. It invites collaboration among surgeons, anesthesiologists, pain management specialists, nutritionists, physical and occupational therapists, and all other members of the care team in the dynamic optimization of perioperative care [85,89]. The integration of physical therapy and rehabilitation services to support patient recovery and optimize functional outcomes is paramount. This is especially important in the postoperative course to mitigate pain and facilitate early mobility [84]. 

Overall, ERAS protocols are associated with improved patient outcomes in minimally invasive spine surgery. They play a key role in the reduction in length of hospital stay, postoperative pain, complication rates, and improved patient satisfaction [85]. Yet, despite evidence of the effectiveness of ERAS protocols in various surgical fields and the increasing implementation of them in MIS practice, there is not a universally agreed upon ERAS protocol for MIS spine surgery [90]. Although the existing MIS ERAS protocols are heterogeneous with respect to drug regimens, they are uniform in their aim of limiting opioid usage, incorporating multimodal pain management, encouraging early ambulation, and early advancement of diet [85]. 

### 6.4. Digital Care Pathways

While minimally invasive surgery has reduced the surgical footprint and subsequently the length of postoperative hospital stays [45], this has led to an increased reliance on patient support systems to help patients as they recover and ease them back into their activities of daily living. Given the ubiquitous use of smart phones and internet-accessing devices, there have been early efforts to provide patients with pre- and postoperative educational materials and tools to help them learn more about their condition, surgery, and recovery. During the postoperative period, readily accessible digital aids may provide value to this unique population and represent the culmination of patient-centered care by contextualizing and correlating subjective patient-reported outcome measures with objective data.

One of the first descriptions of a digital care implementation into the postoperative spine surgery protocol was by Ponder et al., through an application called ManageMySurgery (MMS) [91]. Through this application, the research team was able to collect patient-reported outcome measures, offer self-monitoring features, answer frequently asked questions, and provide educational materials on the patient’s surgery [91]. In a larger follow-up study of over 1000 patients, Venkatraman et al. found that spine surgery patients making use of this app experienced fewer 90-day readmissions and were 32% less likely to visit the emergency room within 90 days following their surgery [6]. Although these tools are cost-effective means to provide patients with information and collect data, it is important that patients are not overly reliant on such technology and delay presentation for a concerning postoperative development.

An emerging area of innovation concerns health tracking technology, which has recently become increasingly common in the age of the smart phone. One such study attempted to correlate objective data collected from the Apple Health mobile application to subjective patient-reported outcomes [92]. In this study, Chauhan et al. found that patients who reported subjective improvement in pain concomitantly exhibited significant increases in physical activity [92]. Other, larger studies have similarly correlated movement scores with patient VAS and ODI [93,94]. Taken together, these findings suggest that smartphone-derived mobility data can effectively complement traditional patient-reported outcome measures, providing a more objective measure of functional outcomes post-surgery. Moreover, preoperative mobility tracking may provide additional data points to the previously described preoperative optimization protocols and calculators to further enhance the care of the surgical spine patient.

## 7. Conclusions

In summary, the integration of preoperative optimization and artificial intelligence into spine surgery risk stratification holds promise for enhancing preoperative planning and patient selection. While advancements in imaging technology, regional anesthesia, and awake spine surgery have transformed the minimally invasive surgical care of the perioperative spine patient, challenges remain. Finally, postoperative care is undergoing notable advancement, all in an attempt to enhance patient care. Continued research and collaboration are essential to address current challenges and further optimize perioperative patient care in minimally invasive spine surgery.

## Data Availability

Not applicable. No new data were created or analyzed in this study. Data sharing is not applicable to this article.
